# A perivascular epithelioid cell neoplasm of the bladder diagnosed after six years: A case report

**DOI:** 10.1016/j.eucr.2025.103228

**Published:** 2025-10-01

**Authors:** Ji-yao Yang, Xiao-rong Yang, Wei-xiang Hei, Yi-dao Liu

**Affiliations:** Department of Urology, Dehong Hospital Affiliated of Kunming Medical University (Dehong Prefecture People's Hospital), Mangshi, Yunnan, China

**Keywords:** Perivascular epithelioid cell neoplasm, Bladder

## Abstract

Perivascular epithelioid cell neoplasm (PEComa) is a rare mesenchymal-derived tumor with specific histological and immunohistochemical manifestations. Given the rarity of bladder PEComa, standardized diagnostic and therapeutic protocols have yet to be established. This article presents a case of bladder PEComa that was ultimately diagnosed after a six-year delay and reviews recent studies to improve clinicians' understanding of this tumor and minimize the risk of misdiagnosis.

## 1 Introduction

The bladder's perivascular epithelioid cell neoplasm (PEComa) is a rare tumor in the urinary system. While PEComa is more commonly found in the kidneys, its occurrence in the bladder is extremely rare, with fewer than 40 cases reported in the literature. PEComa is a mesenchymal-derived tumor with distinct histological and immunohistochemical features. Due to its rarity, it is often misdiagnosed as bladder cancer based on preoperative imaging, as no standardized diagnostic or treatment protocols are currently available. This article presents a case of bladder PEComa, diagnosed after a six-year delay. It reviews recent studies to enhance clinicians' understanding of this tumor and reduce the risk of misdiagnosis.

## 2 Case presentation

**Fig. 1:**
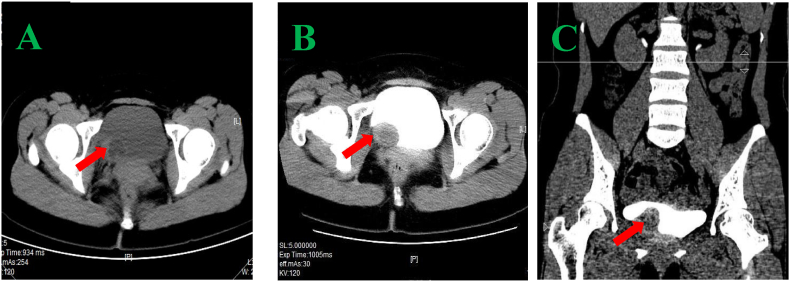
CT image (A–C): A pelvic cystic-solid nodular shadow (33 mm × 27 mm) with a clear boundary. The enhancement scan shows noticeable enhancement of the cystic wall and wall nodule, low density within the cyst, and no thickening of the adjacent bladder wall.

**Fig. 2:**
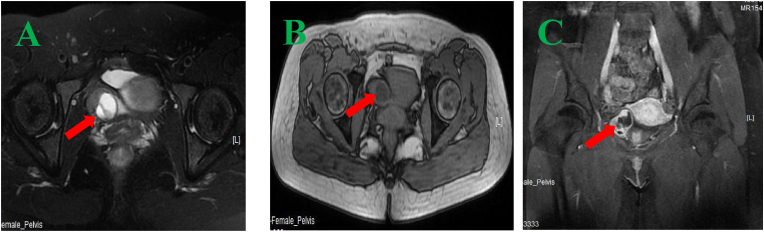
MRI image (A–C): A cystic-solid mass (31.9 mm × 24.5 mm × 37.4 mm) located in the right side of the pelvis (between the right ovary, uterus, and bladder), with enhancement of the solid part observed on the enhanced scan.

**Fig. 3:**
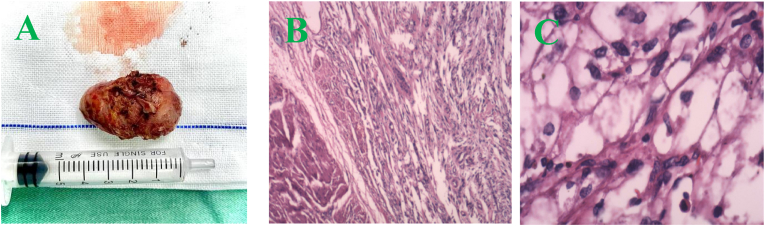
A: Specimen; B (HE × 4): Tumor cells interspersed between smooth muscles of the bladder wall; C (HE × 40): Nested tumor cells with translucent cytoplasm.

**Fig. 4:**
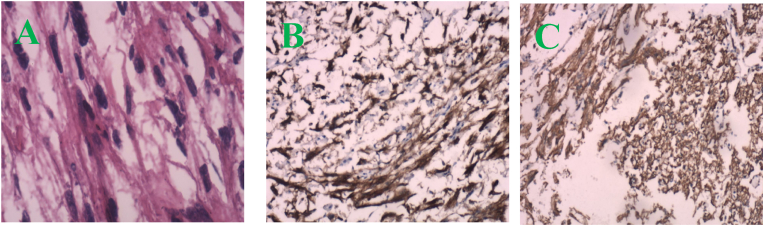
A (HE × 40): Tumor cells arranged in bundles; B (HE × 10): HMB-45(+); C (HE × 10): SMA(+).

A 31-year-old woman was diagnosed with bladder PEComa after a six-year delay. In June 2016, ultrasound imaging detected a solid hypoechoic protrusion (15 mm × 19 mm) on the right posterior bladder wall, but cystoscopy showed no abnormalities. Regular follow-up examinations revealed progressive tumor growth, with a hypoechoic mass (26 mm × 22 mm) observed in May 2021 and a cystic mass (34 mm × 21 mm) associated with the bladder in February 2022. CT imaging revealed a cystic-solid pelvic lesion (33 mm × 27 mm) with clear boundaries. Contrast-enhanced imaging showed significant enhancement of the cystic wall and wall nodule, with low-density areas within the cyst and no thickening of the adjacent bladder wall (Fig. 1A–C). MRI identified a cystic-solid pelvic mass (31.9 mm × 24.5 mm × 37.4 mm) located between the uterus, ovary, and bladder (Fig. 2A–C). In June 2022, laparoscopic resection of the pelvic mass and partial cystectomy were performed. During the procedure, the tumor was found in the right bladder wall, adhering to the bladder wall muscle and mucosal layers. Immunohistochemical staining revealed HMB-45 (partially positive), SMA (positive), and Melan-A (partially positive), confirming the diagnosis of benign PEComa (Fig. 3, Fig. 4). Surveillance with annual pelvic CT scans over 24 months has shown no evidence of recurrence. The final histopathology confirmed a benign PEComa with negative surgical margins. Based on these findings, adjuvant therapy was deemed unnecessary. Genetic testing for TSC or TFE3 rearrangements was not performed due to financial constraints.

## 3 Discussion

Reviewing the clinical manifestations of bladder PEComa reported to date, most patients present with nonspecific symptoms such as ‘lower abdominal distension, pain, and hematuria’. When combined with imaging findings, these symptoms often lead to misdiagnosis as bladder cancer.^1^ In contrast, the patient in this case exhibited no significant clinical signs, which further complicated the diagnosis.

Bladder PEComa must be differentiated from bladder cancer, paragangliomas, and ovarian-related cysts in women.^2^ Cystoscopy is a valuable tool for patients with imaging findings suggestive of a bladder tumor. In this case, cystoscopy revealed a well-defined, submucosal lesion without mucosal disruption or invasive features, consistent with PEComa. The utility of cystoscopic biopsy remains uncertain, particularly for PEComa, as tumors are often located in the submucosal or muscular layers, making it challenging to obtain adequate tissue through biopsy. This underscores the limitations of cystoscopic biopsy in diagnosing bladder PEComa. In this case, after discussing the potential risks of repeat cystoscopy or biopsy (e.g., bleeding, nondiagnostic sampling due to the submucosal location) against the advantage of definitive resection, the patient consented to laparoscopic surgery, which achieved both diagnostic and therapeutic goals. For female patients with suspected exophytic bladder PEComa, Zeno et al. concluded that transvaginal ultrasound can accurately localize extravesical PEComa and evaluate the involvement of surrounding tissues.^3^ Additionally, patients with paragangliomas may experience elevated blood pressure and palpitations during voiding, which are not observed in PEComa. In female patients, the cystic appearance of PEComa can also mimic ovarian cystadenomas, which typically present as watery densities with mild to moderate enhancement on CT.^2^

In this case, the decision to perform laparoscopic partial cystectomy was based on the tumor's location on the outer bladder wall, its abundant blood supply, and its firm adhesion to both the muscular and mucosal layers, which made complete resection via TURBT impossible. Laparoscopic surgery not only effectively removes the tumor but also significantly reduces the risk of intraoperative bleeding. Surgical intervention remains the primary treatment approach for bladder PEComa. The choice between partial cystectomy and ERBT depends on the tumor's size, location, and depth of invasion.^1^^,^^3^ Given the rarity of bladder PEComa, a standardized surveillance protocol is lacking. We recommend initial imaging follow-up (e.g., CT/MRI every 6–12 months), particularly for tumors with atypical features. mTOR pathway inhibitors may offer therapeutic benefits in malignant cases, although their efficacy may be limited in the presence of concomitant TFE3 gene rearrangements.^4^ Genetic testing and multimodal therapy are essential for managing malignant bladder PEComa. Although not obtained in this case, genetic testing for TFE3/TSC holds prognostic and therapeutic utility, especially for malignant variants.

## 4 Conclusions

In conclusion, bladder PEComa is a rare genitourinary tumor that primarily affects younger individuals, with a female predominance. The clinical presentation is often nonspecific, and imaging findings are non-distinctive, necessitating histopathological confirmation for a definitive diagnosis. While typically benign, a subset demonstrates malignant behavior. Surgical resection, ranging from partial cystectomy to ERBT based on tumor characteristics, remains the mainstay of treatment. For malignant cases, mTOR inhibitors may offer therapeutic value, though TFE3 gene rearrangements can limit efficacy. Genetic testing and multimodal management are essential in malignant variants.

## CRediT authorship contribution statement

**Ji-yao Yang:** Writing – review & editing, Writing – original draft, Visualization, Software, Resources, Methodology, Investigation, Funding acquisition, Formal analysis, Data curation, Conceptualization. **Xiao-rong Yang:** Validation, Resources, Investigation, Data curation, Conceptualization. **Wei-xiang Hei:** Investigation, Data curation. **Yi-dao Liu:** Supervision, Project administration, Funding acquisition, Formal analysis, Conceptualization.

## Ethics statement

Written informed consent was obtained from the patients for the images and medical history information published herein.

## Funding

The authors declare financial support was received for this article's research, authorship, and/or publication. This work was supported by the Scientific Research Fund Program of Dehong Prefecture People's Hospital (NO. 2023DY004); the Scientific Research Fund Program of Dehong Prefecture People's Hospital (NO. 2025DY006); FY2023 Special Self-funding for Science and Technology Program Projects in Dehong (NO. ZC202319); FY2023 Special Self-funding for Science and Technology Program Projects in Dehong (NO. ZC202317).

## Conflict of interest

The authors declare that the research was conducted in the absence of any commercial or financial relationships that could be construed as a potential conflict of interest.
